# Does Timing of Colon Procedures Affect Outcomes in D-IBS Trials?

**DOI:** 10.4021/gr238e

**Published:** 2010-09-20

**Authors:** Jianmin Wang, Beth Sherrill, Lynne A. Hamm, Allen W. Mangel

**Affiliations:** a RTI Health Solutions, Research Triangle Park, NC, USA

**Keywords:** Irritable bowel syndrome, Colonoscopy, Symptom scores, Diarrhea-predominant, Pain, Symptoms

## Abstract

**Background:**

Sigmoidoscopy/colonoscopy is usually performed prior to enrollment into clinical trials of irritable bowel syndrome (IBS). Two main reasons are to rule out alternative diagnoses and to ensure that colitis is not present. However, the possible impact of a recent versus remote colon procedure on symptoms in IBS trials has not been evaluated. The aim of this study was to evaluate the effect of timing of colon procedures on symptoms in IBS trials.

**Methods:**

Post hoc analyses were conducted using placebo patients with diarrhea-predominant IBS in a phase 2 trial. Pain, frequency, consistency, and urgency were analyzed using repeated measures models during the first 7 days of treatment and over the entire 12-week treatment period.

**Results:**

Fifty-two placebo patients were grouped by whether they had a colon exam performed between screening and randomization (Group 1) or had a normal colon procedure during the 3 years prior to screening for this trial (Group 2). Average screening symptom scores were comparable between the two groups. Evaluation of various symptoms showed that there were no consistent significant differences between the two groups in pain, frequency, consistency, or urgency.

**Conclusions:**

After the required 3-day post-procedure recovery period, there was no evidence that colonoscopy timing affected subsequent IBS symptoms.

## Introduction

As part of the entry criteria for most clinical trials of irritable bowel syndrome (IBS), patients have an evaluation of their colonic mucosa by colonoscopy or flexible sigmoidoscopy. This evaluation serves two purposes: to ensure that an alternative diagnosis, such as an inflammatory bowel disease, may not account for the symptoms presumed to be related to IBS; and to determine whether preexisting colitis is present in the patients, such that if rectal bleeding were to occur during the study, a drug would not be falsely labeled as the cause. Colitis is a very sensitive safety issue in IBS trials as it has been the cause for lack of approval or withdrawal from the market of several IBS drugs [1, 2]. The timing of a previously performed colonoscopy or flexible sigmoidoscopy is variable from study to study, but usually they must have been conducted within 2 - 3 years of study initiation or the procedures need to be repeated prior to patients participating in a new study.

A question that has not been addressed is: what is the duration of impact of a colon procedure on patient symptoms in IBS trials? The United States Food and Drug Administration (FDA) recently asked this particular question of a pharmaceutical company. Although it may seem intuitive that there are no long-lasting effects of a colon procedure on patients’ symptoms, this question warrants proper evaluation. In the present study, we evaluated existing data from an IBS clinical trial and compared symptoms between patients who had colon examinations just prior to treatment initiation versus patients who had colon evaluations in the more remote past.

## Methods

### Study design and patient population

The data source was a randomized, double-blind, multicenter, phase 2 IBS study conducted in 120 centers in the United States that evaluated efficacy and safety of a novel drug compared with placebo (ClinicalTrials.gov identifier: NCT00454688). The study was approved by an institutional review board, and informed consent was obtained from all patients prior to screening. Subjects could be male or female with at least 6 months of symptoms who met the Rome II criteria for IBS [3].

The study began with a 2-week screening phase during which subjects recorded daily self-assessments of abdominal pain or discomfort and gastrointestinal (GI) symptoms using a touch-tone telephone data entry system. To be eligible for the study, subjects must have reported during screening an average abdominal pain or discomfort score between mild and moderate severity; at least mild pain on at least 3 days over each of the 2 weeks during the screening period; and at least one bowel movement over each week of screening. Furthermore, the subjects had to have a record of normal results from a colon exam after the onset of their IBS symptoms and within the past 3 years (flexible sigmoidoscopy if aged less than 50 years; colonoscopy or a barium enema plus flexible sigmoidoscopy if aged at least 50 years.) If the patients did not have a colon exam within that 3-year period, then a colon exam was performed after the screening period within 4 days of satisfying symptom criteria. For patients whose symptoms had changed since their last procedure, the procedure was repeated irrespective of when it was performed. After the colon examinations, 3 days of recovery were allowed prior to randomization and initiation of therapy.

Self-assessment of symptoms continued daily during the 12-week treatment phase. Abdominal pain or discomfort data were collected daily on a 4-point Likert scale (0 = none; 1 = mild; 2 = moderate; 3 = severe). Subjects recorded daily stool frequency and whether they experienced a sense of urgency to go to the bathroom (yes or no). Consistency was rated using the 7-point Bristol Scale [4]: 1 = separate hard lumps, like nuts, hard to pass; 2 = sausage shaped but lumpy; 3 = like sausage but with cracks on its surface; 4 = like sausage or snake, smooth and soft; 5 = soft blobs with clear-cut edges (passed easily); 6 = fluffy pieces with ragged edges, a mushy stool; 7 = watery, no solid pieces.

### Statistical analysis

This exploratory analysis focused on patients with diarrhea-predominant IBS (IBS-D) who received placebo treatment. We used only placebo patients, versus those receiving active treatment, to avoid confounding effects of the therapeutic agent. Patients were grouped by whether they had a colon exam performed in the interval between the end of screening and randomization (Group 1) or within the 3 years prior to study initiation (Group 2). Patient and disease characteristics were summarized by these groups; patient average screening symptom scores were used as baseline values in analyses.

Response variables included daily pain scores, number of bowel movements (BM), stool consistency, and urgency. All variables were collected daily on the touch-tone telephone data entry system. The proportional odds model was used to analyze daily pain scores (0 - 4) as an ordered response variable. The BM frequency and consistency scores were treated as continuous variables and analyzed using linear mixed models. Urgency was a dichotomous response variable analyzed with logistic regression. In each case, the models were constructed with repeated measurements for daily responses with patient-specific random intercepts and covariates for average screening symptom values.

Models were first run on daily scores for the first week of treatment to evaluate whether any differences between groups occurred during the beginning of the treatment period. Overall differences were tested using a joint test (7 degrees of freedom) for whether the daily difference in average values between the groups was significantly different from zero. Next, models were run across the entire 12-week treatment period; interaction effects of score by time were tested. Where the joint test of weekly effects (12 degrees of freedom) was significant, we comment on group differences in weekly scores.

## Results

### Patient characteristics

Among 583 patients who were enrolled in the study and answered at least one efficacy question, 147 were randomized to receive placebo and 52 of these had IBS-D. The subgroup was fairly evenly divided by whether patients had a colon exam performed after screening (Group 1, N = 28) or within the past 3 years prior to study initiation (Group 2, N = 24). Baseline characteristics were comparable between Groups 1 and 2, with the exception of a longer time since IBS diagnosis for patients in Group 2 (Table 1).

**Table 1:** Summary of Baseline Characteristics for IBS-D Patients Taking Placebo

Characteristic	Group 1 (n = 28)	Group 2 (n = 24)
Age (year), mean (sd)	46.9 (14.9)	49.7 (14.1)
Race (% white), n (%)	27 (96%)	22 (91%)
Gender (% female), n (%)	19 (68%)	18 (75%)
Height (cm), mean (sd)	167.7 (8.2)	166.0 (9.0)
Weight (kg), mean (sd)	76.2 (13.6)	77.9 (16.2)
Years since onset of IBS symptoms, mean (sd)	13.9 (12.7)	13.2 (14.9)
Years since IBS diagnosis, mean (sd)	2.3 (2.4)	4.2 (9.0)
Full colonoscopy (instead of sigmoidoscopy), n (%)	25 (89%)	23 (96%)
Days between colon procedure and first dose of placebo, mean (sd)	4.9 (2.2) Range: 1 - 3	506 (329) Range: 35 - 1160

Average screening symptom scores were comparable between Groups 1 and 2, and this would be anticipated as colon examinations occurred after collection of these symptoms in Group 1 (Table 2). In both groups, mean pain scores during screening were approximately 2 and average consistency on the Bristol Scale represented a soft blob. Mean number of daily bowel movements was 3.15 among patients in Group 1 and 3.95 for patients in Group 2 (P = 0.10). On average, patients in each group experienced urgency on approximately 81% of the screening days, consistent with previous reports that IBS-D patients have urgency as one of their main bowel disturbances when their IBS is active [5]. After meeting the screening criteria, patients in Group 1 had the colon procedure and recovered for an average of 4.9 ± 2.2 days before starting placebo treatment.

**Table 2:** Summary of Symptoms During Screening Period

Symptom	Group 1 (n = 28) Mean (sd)	Group 2 (n = 24 ) Mean (sd)	P Value
Pain score	2.07 (0.39)	2.12 (0.46)	0.327
Number of bowel movements	3.15 (1.25)	3.95 (2.33)	0.100
Consistency	5.07 (0.85)	5.28 (1.00)	0.371
Percentage of screening days with urgency	80.6 (22.7)	80.8 (19.9)	> 0.999

### Relationship between colonoscopy and symptoms

**Figure 1:**
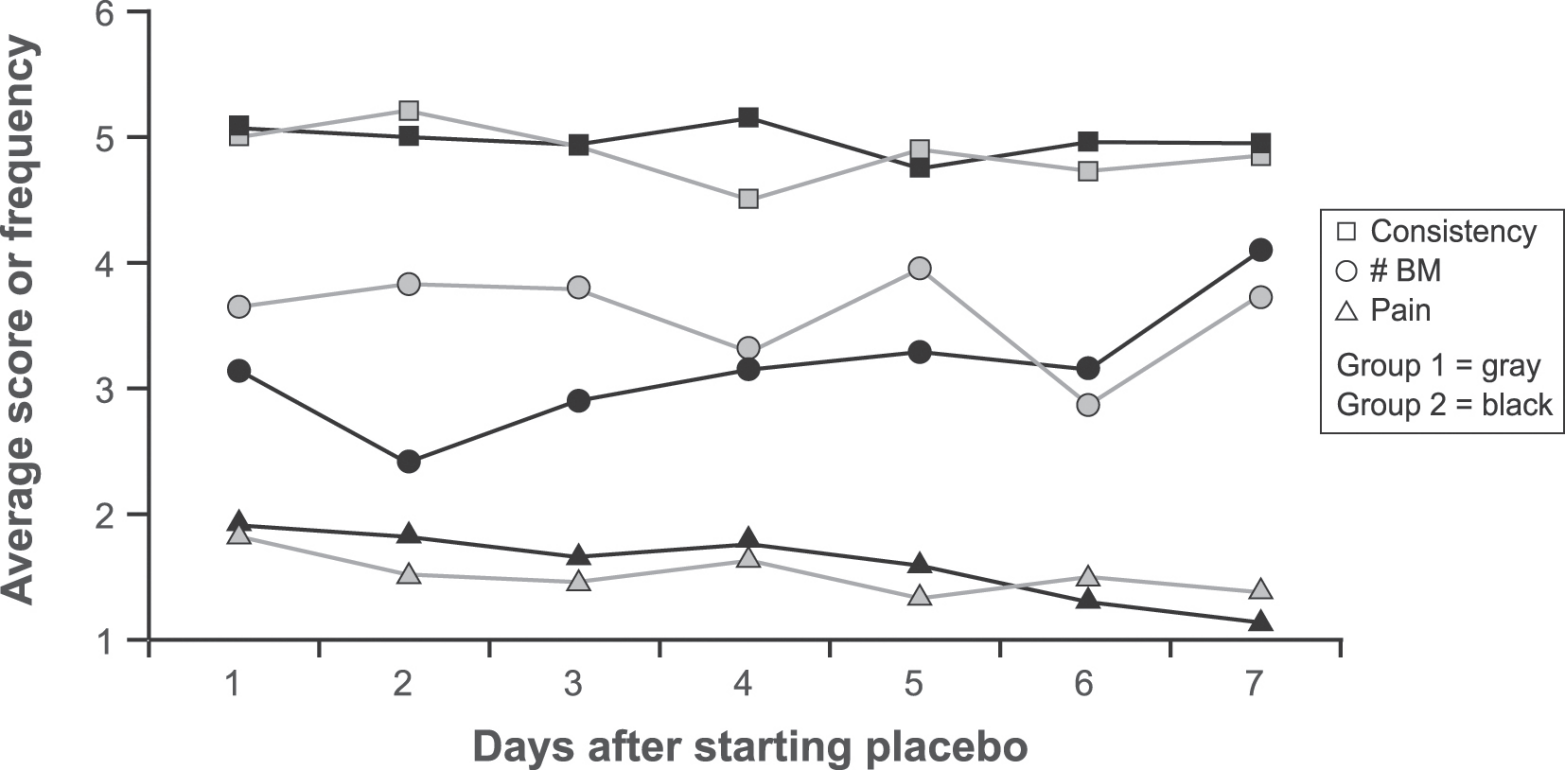
Average daily stool consistency score, frequency and pain score by group during the first 7 days of treatment. Abbreviations: BM, bowel movement.

**Figure 2:**
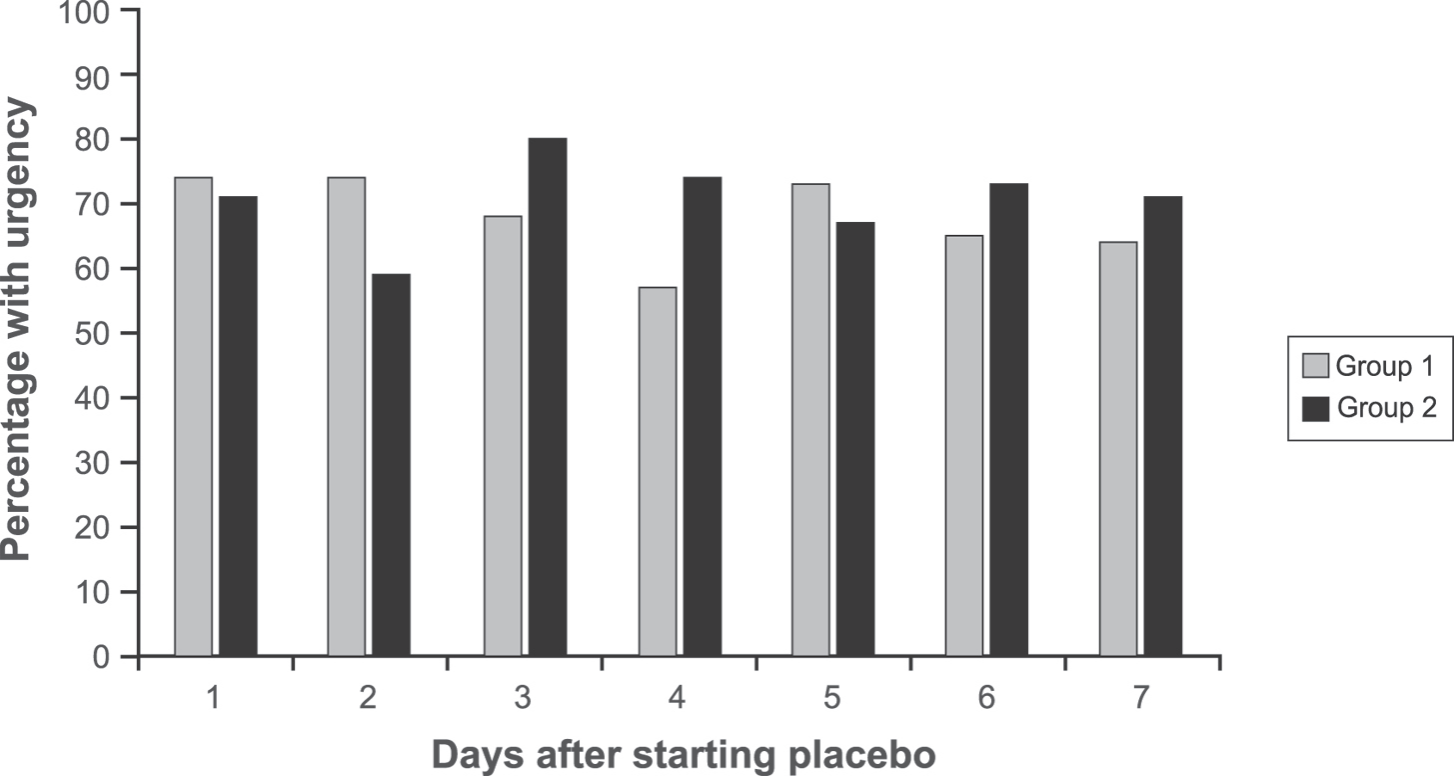
Percentage of patients with urgency by group during the first 7 days of treatment.

During the first 7 days of placebo treatment, no significant differences between groups were noted for daily pain scores (P = 0.72), number of BM (P = 0.20), consistency (P = 0.29), or urgency (P = 0.44) in models that adjusted for baseline symptom scores (Fig. 1, 2).

Both groups had declines from baseline in average symptom scores during the 12-week study period (Table 3). There were no significant differences between groups in pain scores overall (P = 0.47) or at any given week. While most weekly differences were not significant for bowel movement frequency (overall P = 0.074), at week 7 the average decline in number of bowel movements per day was significantly greater in Group 2 than in Group 1 (P = 0.014). However, in week 8, the reduction from baseline in Group 1 bowel frequency was similar to that in Group 2 and the difference was not significant. The overall test for consistency differences between groups was significant (P = 0.003), most of which was attributable to higher average scores in Group 1 (representing less than one point on the Bristol scale) at weeks 7, 8, and 9. No meaningful clinical differences in group consistency scores occurred for weeks 10 - 12 or for weeks 1 - 6. Declines in percentage of days with urgency were not statistically different between groups overall (P = 0.99) or at any week.

**Table 3:** Summary of Symptom Changes^a^ From Baseline During 12-week Treatment Period

Symptom	Week	Group 1 (n = 28)	Group 2 (n = 24)	P Value^b^
Mean change from baseline in pain score	1	-0.528	-0.544	0.966
	2	-0.703	-0.619	0.968
	3	-0.767	-0.805	0.488
	4	-0.644	-0.767	0.150
	5	-0.789	-0.639	0.860
	6	-0.895	-0.571	0.662
	7	-0.948	-0.872	0.506
	8	-0.845	-0.881	0.361
	9	-0.923	-0.916	0.431
	10	-1.055	-0.834	0.901
	11	-0.991	-0.909	0.659
	12	-0.948	-0.874	0.973
Mean change from baseline in number of bowel movements	1	-0.084	-0.428	0.379
	2	-0.192	-0.650	0.299
	3	-0.138	-0.583	0.187
	4	-0.264	-0.751	0.122
	5	-0.309	-0.280	0.765
	6	-0.338	-0.390	0.686
	7	-0.161	-1.072	0.014
	8	-0.308	-0.517	0.628
	9	-0.078	-0.686	0.127
	10	-0.114	-0.450	0.403
	11	-0.294	-0.641	0.290
	12	-0.276	-0.111	0.577
Mean change from baseline in consistency	1	-0.118	-0.492	0.173
	2	-0.400	-0.254	0.377
	3	-0.370	-0.539	0.507
	4	-0.142	-0.506	0.086
	5	-0.284	-0.149	0.833
	6	-0.422	-0.444	0.487
	7	-0.278	-0.710	0.047
	8	-0.139	-0.607	0.049
	9	-0.178	-0.772	0.010
	10	-0.558	-0.573	0.706
	11	-0.301	-0.476	0.238
	12	-0.474	-0.305	0.661
Mean change from baseline in percentage of days with urgency	1	-0.102	-0.126	0.8194
	2	-0.147	-0.174	0.6878
	3	-0.150	-0.250	0.6348
	4	-0.199	-0.227	0.6007
	5	-0.166	-0.176	0.8077
	6	-0.211	-0.198	0.8531
	7	-0.162	-0.266	0.0571
	8	-0.129	-0.299	0.1459
	9	-0.226	-0.321	0.2236
	10	-0.254	-0.303	0.3325
	11	-0.263	-0.289	0.8031
	12	-0.210	-0.318	0.1737

a Values shown are group mean changes from the screening period in unadjusted symptom scores.

b P value from test of group difference at each week from repeated measures model adjusted for baseline value.

## Discussion

Evaluation of potential therapeutic agents to be used for the treatment of IBS has revealed several relevant confounding factors. For example, alosetron, a drug indicated for the treatment of D-IBS patients, shows efficacy only in female patients [5, 6], and it is efficacious in D-IBS patients but not in patients with IBS with alternating diarrhea and constipation (A-IBS) [5-7]. Tegaserod, which was approved in the United States for treatment of only one IBS subtype, constipation-predominant IBS (C-IBS), was also shown to be efficacious only in female patients [8]. Thus, when consider evaluations of potential agents for the treatment of IBS, relevant subgroups, for example, gender and IBS subtype, need to be considered.

An interesting additional factor to consider has also recently emerged in IBS trials, entry baseline pain. In phase 2b trials with the kappa opioid agonist asimadoline, D-IBS patients with at least moderate pain during the screening or baseline period showed efficacy while those with milder pain did not [9]. As the high unmet medical need for IBS resides in the moderately to severely affected population, this is a positive finding for a drug to be efficacious in the more afflicted patients.

In the present study, we evaluated whether timing of prerandomization colon evaluations affected IBS symptoms in placebo patients during the treatment period. A limitation of these exploratory analyses is that they were conducted on a subgroup of D-IBS patients receiving placebo, and patients were not randomized based on timing of colon procedures. However, the groups showed comparability on IBS symptoms collected during the screening period prior to the colon procedure and there is no reason to suspect inherent bias. The comparison of placebo patients who had recent versus past colon evaluations revealed no differentiation in IBS symptoms during the first 7 days of treatment. Both groups declined in pain, frequency, consistency, and urgency during the 12-week treatment period, with small differences between groups observed approximately 2 months after baseline. During weeks 7 - 9 of evaluation, significantly greater reduction in stool frequency and improvement in stool consistency was seen in Group 2 than in Group 1. However, given the cyclical nature of IBS and the fact that no differences were seen until this late time point, we conclude that the timing of the colon examination did not affect overall efficacy assessments. Thus, specific analyses relating to timing of colon evaluations should not be required as part of analytical plans in IBS clinical trials.
